# Influence of Vision on Drivers: A Pilot Study

**DOI:** 10.3390/ijerph182212116

**Published:** 2021-11-18

**Authors:** Cristina Alvarez-Peregrina, Clara Martinez-Perez, Cesar Villa-Collar, Miguel Ángel Sánchez-Tena

**Affiliations:** 1 Faculty of Biomedical and Health Science, Universidad Europea de Madrid, 28670 Madrid, Spain; cristina.alvarez@universidadeuropea.es (C.A.-P.); villacollarc@gmail.com (C.V.-C.); 2 ISEC LISBOA—Instituto Superior de Educação e Ciências, 1750-179 Lisboa, Portugal; masancheztena@ucm.es; 3 Department of Optometry and Vision, Faculty of Optics and Optometry, Universidad Complutense de Madrid, 28037 Madrid, Spain

**Keywords:** vision, driving, Spain

## Abstract

Background: Driving is the main mode of transportation in many countries, and visual safety depends largely on good visual health. The objective of this study is to analyze the visual health of Spanish drivers; as well as analyze the difference between professional and non-professional drivers. Methods: A visual screening was carried out in Spanish drivers from all over Spain, in which the following tests were performed: monocular visual acuity in distance and near vision, visual field, stereopsis, contrast sensitivity, intraocular pressure and balance test binocular. Subsequently, a questionnaire was carried out on the patient’s driving data and ocular antecedents. Results: 74.5% of the drivers used glasses to drive, of which 61.5% used progressive glasses. However, 39.4% reported having difficulties seeing well. The mean visual acuity in the distance and near was 0.93 ± 0.13 and 0.94 ± 0.13, respectively. Significant differences have been found in accident risk based on visual acuity (*p* < 0.001). But no significant differences have been found in terms of visual field, stereopsis, contrast sensitivity, binocular balance and intraocular pressure (*p* > 0.05). Conclusion: Vision appears to play a key role in driving and a good visual assessment is recommended for early detection of visual problems that may affect road safety. A study with a larger sample size would be necessary to confirm the results of this pilot study.

## 1. Introduction

Driving is a visual task which includes several sensory visual functions [1]. Therefore, an adequate vision is vital to ensure a safe driving. It is estimated that up to 90% of the information we need to have a safe drive comes from the visual system [2]. The visual search capacity is one of the most important skills to enjoy safe driving [3]. During this process, several attention methods are used to remember traffic elements, such as recognising objects or remembering searched and non-searched elements. Thus, other skills are also required in order to ensure a safe driving, such as mental skills, motor skills and compensatory skills [1].

The visual acuity testing (VA) is one of the most important and common tests to assess the visual function. That is to say, it is the most widely used criterion in order to determine the driving ability. For this reason, in most countries, it is necessary to take a visual screening to obtain the driving license. However, these strategies may not accurately measure the skills that are necessary to enjoy safe driving [4]. It happens because the Snellen chart is not equivalent to the “number plate test”, and many eye care professionals consider that a binocular VA of 20/32 Snellen meet the driving standards while patients with a binocular VA of 20/40 Snellen don’t [5]. In turn, there is not any common standard regarding the required VA within the different countries and the most common standard threshold is 20/40 Snellen [6]. Besides, the specific requirements to assess the field of vision also vary from country to country [1]. The most common way of travelling in many countries, both for leisure and for work, is driving; and in some rural areas it is the only alternative available [7]. Therefore, for elderly people and for people who suffer from mobility problems, driving may be the only option they have to feel independent, to have some social interaction and to enjoy a better quality of life [8]. Elderly people are the segment of population with the quickest worldwide growth. In Spain, it is estimated that, by 2050, people over 65 years old would account for 30% out of the total population; thus, increasing the number of older drivers [9,10]. In the last driver register conducted by the General Directorate of Traffic in 2019, people over 65 years old accounted for 16% of the total number of Spanish drivers [11]. In comparison with young drivers, these older drivers have a higher risk of suffering an accident due to a decrease in visual function, visual processing, their cognitive abilities and an increase in the prevalence of visual pathologies [8,12,13,14]. This happens because ocular changes related to ageing, such as the clouding of the crystalline lens, lead to an increase on the intraocular light scattering that creates a diffuse veil of light in the retina, which causes a disabling glare [15]. Therefore, although these drivers may have a good visual acuity, they also have vision problems that compromise a safe driving (recognition problems, blurry vision, colour and contrast losses, and backlit vision problems), especially under night vision conditions or when there isn’t enough sunlight [16,17]. In turn, in Spain, the rural population has poorer visual health. This is due to social and economic factors, which cause them to use less optical correction and more eye pathologies, compared to the general population [18].

To reduce the risk of accidents, in recent years, thanks to advances in artificial intelligence, systems based on vision and computers have been developed that allow traffic monitoring, obstacle detection or speed control, among others. Thus, for example, the V2X paradigm is essentially based on the exchange of information in the form of vehicle to infrastructure, vehicle to vehicle, vehicle to pedestrian, vehicle to self and vehicle to roadside units. However, developing high-precision traffic flow prediction algorithms using conventional traffic flow estimation techniques is still a major challenge [19].

This study aims at analysing Spanish drivers’ visual health in order to know its influence on driving and the increase in the risk of accidents. Moreover, it also aims at analysing the difference between professional and non-professional drivers.

## 2. Materials and Methods

### 2.1. Data Collection

An observational, prospective, transversal and multi-centre study was designed. It was carried out in 97 optical centres in Spain, which were associated to the “Vision y Vida” Foundation, during the year 2018. Thus, all Spanish drivers who wanted to carry out a vision check could go to one of the opticians associated with the foundation and have it done free of charge. The objective of this campaign was to reduce the risk of traffic accidents. In order to collect the data, a visual screening through a convenience sampling was performed.

During the visual screening, the following tests were included:

- Monocular visual acuity in far and near vision;
- Field of vision;
- Stereopsis;
- Contrast sensitivity;
- Intraocular pressure;
- Binocular balancing test.

Furthermore, they were asked if they need lenses to drive and presbyopic patients that wear progressive lenses were asked if they had any difficulty seeing clearly.

On the other hand, a questionnaire about driving data was carried out (professional driver, driving experience, driving hours during the day and at night, driving records for the last 3 years) and eye problems history of the patient (ocular or systemic diseases, ocular interventions, medication that could affect vision, presence of some of the following symptoms: glare in sunny days, vision difficulties or reflections while driving at night, eye irritation or fatigue at the end of the day, difficulties to see the signposts or signboards on the motorway or avoid driving at night).

### 2.2. Statistical Analysis

The statistical analysis was carried out using the computer programme SPSS 27.0 (SPSS Inc., Chicago, IL, USA). The normal distribution of the variables was performed using the Kolmogorov–Smirnov test, with a 0.05 significance level. The quantitative variables were described using the mean ± of standard deviation (SD), or the interquartile median and range [IQR], according to the distribution.

In order to compare the study cohorts “professional drivers” and “non-professional drivers”, the Chi-square test was used to measure the qualitative variables; and the Mann–Whitney U or Kruskal–Wallis tests were used to measure the quantitative variables. The odds ratio and its 95% confidence Interval were calculated.

### 2.3. Ethics Committee

This research was carried out according to the principles established by the Declaration of Helsinki and it was approved by the ethics committee of the European University of Madrid (CEI-UE), bearing the code CIPI/19/102.

## 3. Results

A total of 744 patients took part in this study, and 8 of them were excluded due to lack of information in their questionnaire. The remaining 736 participants were divided into 153 (20.8%) professional drivers and 583 (79.2%) non-professional drivers.

### 3.1. Demographic Data

Regarding gender distribution, 65.1% of the participants were males and 34.9% were females. The percentage of males in the professional drivers’ category was significantly higher than the percentage of females (91.5% versus 8.5%; *p* < 0.001).

The age of the participants was 46.42 ± 15.25 years old, and the median was 46 years old. Table 1 shows the demographic data from the tested sample.

Regarding vision, 18.2% were taking some medication that affected their vision (13.0% non-professional drivers and 5.2% professional drivers; *p* = 0.017). However, 82.3% (63.3% non-professional drivers; 19.0% professional drivers) consider that they have a good sight while driving, 15.9% (14.1% non-professional drivers; 1.8% professional drivers) consider that they do not have a perfect sight but they can drive properly and 1.8% believe that they do not have a good sight (1.8% non-professional drivers).

As it is shown in Table 2, significant differences were found regarding the presence of symptoms in both groups (*p* < 0.001).

### 3.2. Visual Screening

From the total number of participants, 74.5% wears lenses to drive (13.2% professional drivers; 61.3% non-professional drivers). Amongst them, 61.5% (50.3% non-professional drivers; 11.3% professional drivers) wear progressive lenses that allow them to see the indicators of the vehicle. However, 39.4% (30.4% non-professional drivers; 9.0% professional drivers) stated that they had some difficulties in order to see them properly.

With regard to visual acuity, the mean and standard deviation for far vision was 0.93 ± 0.13 and for near vision was 0.94 ± 0.13. No significant differences were found regarding visual acuity among professional and non-professional drivers (*p* > 0.05). Further, 1.1% of the sample showed a visual acuity lower than 20/63 Snellen, which is the lowest limit in order to obtain a driving license; and 41.4% had a VA lower than 20/125 Snellen. Significant differences were found regarding the risk of having an accident according to visual acuity (*p* < 0.001). Poor visual acuity increases the risk of a traffic accident.

However, no significant differences were found regarding the increase in the accident rate according to the field of vision, stereopsis, contrast sensitivity, binocular balancing and intraocular pressure (*p* > 0.05).

Table 3 shows the proportion of patients that have or not some alternations in the different test that were performed.

## 4. Discussion

We are living in a society where elderly people are the sector with the quickest growth regarding the number of drivers. This has a negative impact on the road safety, since accidents statistics show that older drivers have a higher accident rate, and they are more frequently the “culprits” regarding these accidents [9].

Our study observed that due to regular visual screenings only 0.7% of the drivers had a visual acuity lower than 20/63 Snellen, which allows to have a better control in terms of road safety. The first researchers that analysed the relationship between visual acuity and road safety were carried out by Burg in 1967 and 1968 [20,21], and subsequently, by Hills and Burg in 1977 [22]. These researchers showed that there was not a relationship between a low visual acuity and the risk of having an accident amongst young and middle age drivers. However, they found a weak association amongst older drivers. This possible relationship has also been found in other studies [23,24,25]. Thus, in the study by Ortiz-Peregrina et al. [26], it was found that in older Spanish drivers such as ocular structures were deteriorated, driving performance was affected. In another study, as in our results, it is estimated that 29.5% of Spanish drivers have visual problems, which need to be addressed to improve the prevention of car accidents and road safety for all road users [27]. However, it is worth mentioning that in two cohort studies carried out by Rubin et al. [28] and Cross et al. [29], which had a sufficient sample size (1801 and 3158 participants, respectively) and were well designed, it was not found any relationship between visual acuity and the risk from suffering a road accident. The reason for the difference between these studies is due to multifactorial causes of traffic accidents and the lack of availability of drivers with poor vision due to death in accidents or the cessation of driving.

Regarding the field of vision, several studies have analysed its influence on the risk of having an accident [30,31]. One of the most quoted studies is the one by Johnson et al. [32], in which it was found that the accident and offences rate was the double for drivers who suffer from a severe binocular vision field loss in comparison with those drivers that did not suffer from it. Thus, defects on the homonymous field of vision may cause an inadequate positioning, an alteration in the sense of space, an inconsistent direction and an increase in collisions [33]. On the other hand, other studies found a limited relationship between people with homonymous defects and a complete vision field loss [34,35]. Regarding quadrantanopsia, driving seems to be safer, probably because the length of the involved field is lower [36,37]. Thus, the studies carried out by Wood et al. [34], Elgin et al. [36] and Parker et al. [37] show that 88%, 87.5% and 87% of the drivers were safe, respectively. Wood et al. [34] suggested that this could be due to the fact that these drivers had a greater head movement towards the involved field, so they could watch the lane as widely as possible in order to reduce sudden braking manoeuvres. However, they did not find out whether or not the risk of accidents was higher if the superior or inferior field of vision was affected. It has also been proven that alternations in the peripheral vision have an impact on driving, since these drivers have longer search times, more fixations with a shorter duration and more errors in comparison with drivers who do not suffer from any alterations in their field of vision [38,39]. On the other hand, as well as it happens in our study, other studies did not find, or they did not express, a relationship between a high accident rate and drivers who suffer from some defects in their field of vision [40,41]. These differences may be caused by the definition of impairment in the field of vision, that is to say, according to the degrees that are impaired in the field of vision. Thus, the established standards vary in the different regions, between a field of vision of 120º–140º with both eyes [42]. Other potential factors are the size of the sample, inclusion criteria and the time lapse from the beginning/adjustment period. In turn, if we compare our study to others, we must consider that Spain has some of the most demanding requirements in order to obtain the driving license; thus, only 8.8% of the participants had an altered field of vision in one eye and, for this reason, a relationship with an increase of the accident rate was not found.

Contrast sensitivity is another factor that has an impact on driving. Even though it does not exist as much current literature on contrast sensitivity and road safety as it does about visual acuity, it is not less important. Several studies have analysed their relationship, for example, Rubin et al. [43] assessed the relationship between visual impairment, including contrast sensitivity, and the functional capacity in a sample obtained from American elderly people. They discovered that contrast sensitivity was the most perceived factor amongst drivers, which led to difficulties while driving during the day and at night. In turn, Owsley et al. [44], found out that drivers who have a monocular reduction in their contrast sensitivity were three times more likely to have an accident. However, other studies, as well as ours, did not find a relationship between contrast sensitivity and road safety [28,29]. This could be due to the study population, as in our study the participants’ average age was 46.42 ± 15.25 years old, and therefore, it represents a sector of the population with a lower probability of suffering from visual impairments in comparison. In contrast, other studies have a sample comprised of older drivers, and therefore, the risk of suffering from cataracts would be higher. In turn, a lack of association in the prospective studies may be related to the current laws that exist in each country. That is to say, drivers who have a great contrast sensitivity impairment are less likely to renew their driving license. On the other hand, these drivers may suffer from glare sensitivity; thus, having a negative impact on their driving skills. This skill is crucial, especially when driving at night, when we cannot detect peripheral stimuli around the headlights. This problem may lead to a higher risk of having an accident. Previous studies have reported the same findings, in which an increase in the halo size is linked to greater driving difficulties [45]. Likewise, the study carried out by Kimlin et al. [46] showed a significant relationship between the night driving performance in older drivers and the halo size.

Other factor that has an impact on road safety is the binocular status. Several studies showed that drivers who have a lack of stereoacuity have a higher risk of having a collision, or that the seriousness of the accidents is higher in comparison to drivers who have a normal stereoacuity [47,48]. However, this relationship was not found in other studies, as it happened in ours [28,41]. This can be due to the fact that most of the existing bibliography analyses how binocular vision has an impact on the driver’s capacity to see controls or indicators properly; and not how it has an impact on road safety.

It is worth mentioning that the comparison between professional and non-professional drivers may be not entirely accurate, since professional drivers have a greater driving exposure, and therefore, they are used to heavy traffic conditions. Thus, in the study by Navarro-Valls et al. [49] found that the visual acuity of drivers is approximately 11% better than that of non-drivers in all age ranges, this difference being statistically significant in both photopic and mesopic conditions. This difference may be due to the fact that professional drivers in Spain have to pass more rigorous visual tests and more frequently than non-professionals. However, the results found in this study differ from our results. This difference may be due to the low rate of drivers who have a visual impairment, which could have influenced the results and is therefore a limitation of this study. This is due to the strict visual control that is performed in Spain in order to renew the driving license, so as to decrease the accident rate influenced by vision issues. In turn, basic perceptive biases may also occur since it has been proven that the speed of data processing amongst professional drivers is higher than amongst their non-professional peers [50]. However, in our study, we could not analyse the processing speed because of the reduced sample size regarding professional drivers and the limited age range, which implies a major limitation that will be taken into account in future studies. Neither does the influence of gender on safe driving, due to the high difference in the percentage of participation between both genders. Thus, in the future it is intended to segment the population and analyze information processing components. Another big difference in our study with respect to the study by Gené-Sampedro et al. [51] conducted in Spain this year, was that in their study, unlike our results, drivers showed significantly better test performance than non-drivers. This difference may be due to the fact that in their study they used a test that analyses eye movements specific to adults and in our study a binocular balancing test was used. Therefore, in the future it would be necessary to make a comparison between these two tests to corroborate the differences found in these studies.

## 5. Conclusions

A pilot study is presented that shows the importance of good visual acuity in reducing the risk of accidents. No significant relationship was found between the risk of having an accident and other visual factors (contrast sensitivity, field of vision, binocular vision, etc.). However, a study with a larger sample size would be necessary to confirm the results of this pilot study.

It should be noted that non-professional drivers have more vision problems and eye discomfort when driving than professional drivers.

Early detection of visual problems through screening will help improve road safety. Thus, we recommend that researchers keep working on this in order to raise awareness amongst drivers about the importance of taking care of our visual health, especially amongst older drivers.

## Figures and Tables

**Table 1:** Demographic data from the study population.

	Total	Professional Drivers	Non-Professional Drivers	*p*-Value
**No. of participants** (% of the total)	736	153 (20.8%)	583 (79.2%)	
**Age**				
Mean ± DE	46.42 ± 15.25	42.55 ± 12.58	47.44 ± 15.73	*p* = 0.004
Median (IQR)	46 (22)	44.50 (17)	46 (23)	
**Driving experience** Median (IQR)	1995 (20)	1998 (17)	1994 (20)	*p* = 0.026
**Driving hours**				
Day				
30 min	258 (35.1%)	28 (18.4%)	230 (39.5%)	
1 h	188 (25.6%)	1 (0.7%)	187 (32.1%)	*p* < 0.001
1 h–2 h	108 (14.7%)	4 (2.6%)	104 (17.9%)	
2 h–4 h	62 (8.4)	10 (6.6%)	52 (8.9%)	
4 h–6 h	27 (3.7)	20 (13.2%)	7 (1.2%)	
6 h–8 h	33 (4.5)	32 (21.1%)	1 (0.2%)	
>8 h	58 (7.9)	57 (37.5%)	1 (0.2%)	
Night				
30 min	529 (71.9)	44 (28.8%)	485 (83.2%)	
1 h	79 (10.7)	13 (8.5%)	66 (11.3%)	*p* < 0.001
1 h–2 h	60 (8.2)	37 (24.2%)	23 (3.9%)	
2 h–4 h	38 (5.2)	31 (20.3%)	7 (1.2%)	
4 h–6 h	20 (2.7)	19 (12.4%)	1 (0.2%)	
6 h–8 h	8 (1.1)	7 (4.6%)	1 (0.2%)	
>8 h	2 (0.3)	2 (1.3%)	0 (0.0%)	

SD: standard deviation; IRQ: interquartile range.

**Table 2:** Symptomatology while driving.

	Total	Professional Drivers	Non-Professional Drivers	*p*-Value
Glare in sunny days	170 (25.4%)	47 (7%)	123 (18.4%)	*p* < 0.001
Avoid driving at night	165 (24.7%)	19 (2.8%)	146 (21.9%)	
Vision difficulties or reflections while driving at night	152 (22.8%)	18 (2.7%)	134 (20.1%)	
Eye irritation or fatigue at the end of the day	138 (20.7%)	40 (6.0%)	98 (14.7%)	
Difficulties to see the signposts or signboards on the motorway	43 (6.4%)	4 (0.6%)	39 (5.8%)	

**Table 3:** Visual screening results.

	Total		Professional Drivers		Non-Professional Drivers		*p*-Value	OR (95% CI)
	Normal	Altered	Normal	Altered	Normal	Altered		
**Field of vision** Right eye Left eye	699 (95.0%) 708 (96.2%)	37 (5.0%) 28 (3.8%)	150 (98.0%) 151 (98.7%)	3 (2.0%) 2 (1.3%)	549 (94.2%) 557 (95.5%)	34 (5.8%) 26 (4.5%)	*p* > 0.05	0.323 (0.098–1.066) 0.284 (0.067–1.209)
**Intraocular pressure** Right eye Left eye	711 (96.6%) 711 (96.6%)	25 (3.4%) 25 (3.4%)	148 (96.7%) 149 (97.4%)	5(3.3%) 4 (2.6%)	563 (96.6%) 562 (96.4%)	20 (3.4%) 21 (3.6%)	*p* > 0.05	0.951 (0.351–2.576) 0.718 (0.243–2.125)
**Stereopsis**	681 (92.5%)	55 (7.5%)	146 (95.4%)	7 (4.6%)	535 (91.8%)	48 (8.2%)	*p* > 0.05	0.534 (0.237–1.206)
**Contrast sensitivity**	699 (95.0%)	37 (5.0%)	148 (96.7%)	5 (3.3%)	551 (94.5%)	32 (5.5%)	*p* > 0.05	0.582 (0.223–1.519)
**Binocular balancing**	648 (94.2%)	40 (5.8%)	125 (97.7%)	3 (2.3%)	523 (93.4%)	37 (6.6%)	*p* > 0.05	0.339 (0.103–1.118)

The odds ratio (and its 95% confidence interval) of presenting alterations in each of the visual tests in non-professional drivers with respect to professional drivers.

## Data Availability

“Vision y Vida” foundation.

## References

[1] Irwin C., Mollica J.A., Desbrow B. (2019). Sensitive and Reliable Measures of Driver Performance in Simulated Motor-Racing. Int. J. Exerc. Sci..

[2] Andysz A., Merecz D. (2012). Zdolności wzrokowe starszych kierowców—Przeglad badań symulatoro wych [Visual abilities of older drivers—Review of driving simulator studies]. Med. Pract..

[3] Carsten O.M.J., Brookhuis K. (2005). Issues arising from the HASTE experiments. Transp. Res. Part F Traffic Psychol. Behav..

[4] Patnaik J.L., Pecen P.E., Hanson K., Lynch A.M., Cathcart J.N., Siringo F.S., Mathias M.T., Mandava N. (2019). Driving and Visual Acuity in Patients with Age-Related Macular Degeneration. Ophthalmol. Retina..

[5] Levecq L., Safi A., Jamart J., Potter P.D., Oustabassidis E., Blondeau L., Meeųs L., Van Hollebeke I. (2017). Visual Acuity, Visual Field, and Factors Influencing Automobile Driving Status in One-Thousand Subjects Aged 18 to 59 Years. Int. J. Ophthalmol. Clin. Res..

[6] European Council of Optometry and Optics Visual Standars for Driving in Europe. https://www.ecoo.info/wp-content/uploads/2017/01/Visual-Standards-for-Driving-in-Europe-Consensus-Paper-January-2017....pdf.

[7] Bro T., Lindblom B. (2018). Strain out a gnat and swallow a camel? Vision and driving in the Nordic countries. Acta Ophthalmol..

[8] Hansen S., Newbold K.B., Scott D.M., Vrkljan B., Grenier A. (2020). To drive or not to drive: Driving cessation amongst older adults in rural and small towns in Canada. J. Transp. Geogr..

[9] Ayuso M., Sánchez R., Santolino M. (2020). Does longevity impact the severity of traffic crashes? A comparative study of young-older and old-older drivers. J. Saf. Res..

[10] Fernandez J.L., Parapar C., Ruíz M. El Envejecimiento de la Población. Fundación Genera CSIC. http://www.fgcsic.es/lychnos/es_es/articulos/envejecimiento_poblacion.

[11] Dirección General de Tráfico Tablas Estadísticas. https://www.dgt.es/es/seguridad-vial/estadisticas-e-indicadores/censo-conductores/tablas-estadisticas/.

[12] Connors M.H., Ames D., Woodward M., Brodaty H. (2017). Mild Cognitive Impairment and Driving Cessation: A 3-Year Longitudinal Study. Dement. Geriatr. Cogn. Disord..

[13] Anstey K.J., Wood J.M., Lord S.R., Walker J.G. (2005). Cognitive, sensory and physical factors enabling driving safety in older adults. Clin. Psychol. Rev..

[14] Davis J., Casteel C., Hamann C., Peek-Asa C. (2018). Risk of motor vehicle crash for older adults after receiving a traffic charge: A case–crossover study. Traffic Inj. Prev..

[15] Ortiz-Peregrina S., Ortiz C., Salas C., Casares-López M., Soler M., Anera R.G. (2020). Intraocular scattering as a predictor of driving performance in older adults with cataracts. PLoS ONE.

[16] van den Berg T.J., Van Rijn L., Kaper-Bongers R., Vonhoff D., Völker-Dieben H., Grabner G., Nischler C., Emesz M., Wilhelm H., Gamer D. (2009). Disability Glare in the Aging Eye. Assessment and Impact on Driving. J. Optom..

[17] Ortiz C., Castro J.J., Alarcón A., Soler M., Anera R.G. (2013). Quantifying age-related differences in visual-discrimination capacity: Drivers with and without visual impairment. Appl. Ergon..

[18] Latorre-Arteaga S., Fernández-Sáez J., Gil-González D. (2018). Inequities in visual health and health services use in a rural region in Spain. Gac. Sanit..

[19] Wang J., Shao Y., Ge Y., Yu R. (2019). A Survey of Vehicle to Everything (V2X) Testing. Sensors.

[20] Burg A. (1967). The Relationship between Test Scores and Driving Records: General Findings.

[21] Burg A. (1968). Vision Test Scores and Driving Records: Additional Findings.

[22] Hills B.L., Burg A. (1977). A Reanalysis of California Driver Vision Data: General Findings.

[23] Salvia E., Petit C., Champely S., Chomette R., Di Rienzo F., Collet C. (2016). Effects of age and task load on drivers’ response accuracy and reaction time when responding to traffic lights. Front. Aging Neurosci..

[24] Zhang L., Baldwin K., Munoz B., Munro C., Turano K., Hassan S., Lyketsos C., Bandeen-Roche K., West S.K. (2007). Visual and Cognitive Predictors of Performance on Brake Reaction Test: Salisbury Eye Evaluation Driving Study. Ophthalmic Epidemiol..

[25] Owsley C., Wood J.M., McGwin G. (2015). A roadmap for interpreting the literature on vision and driving. Surv. Ophthalmol..

[26] Ortiz-Peregrina S., Ortiz C., Casares-López M., Castro-Torres J.J., Del Barco L.J., Anera R.G. (2020). Impact of Age-Related Vision Changes on Driving. Int. J. Environ. Res. Public Health.

[27] Lijarcio J.I., Useche S.A., Llamazares J., Montoro L. (2020). Are Your Eyes “on the Road”? Findings from the 2019 National Study on Vision and Driving Safety in Spain. Int. J. Environ. Res. Public Health.

[28] Rubin G.S., Ng E.S.W., Bandeen-Roche K., Keyl P.M., Freeman E., West S.K. (2007). A Prospective, Population-Based Study of the Role of Visual Impairment in Motor Vehicle Crashes among Older Drivers: The SEE Study. Investig. Opthalmol. Vis. Sci..

[29] Cross J.M., McGwin G., Rubin G.S., Ball K., West S.K., Roenker D.L., Owsley C. (2008). Visual and medical risk factors for motor vehicle collision involvement among older drivers. Br. J. Ophthalmol..

[30] Bro T., Andersson J. (2021). The effects of visual field loss from glaucoma on performance in a driving simulator. Acta Ophthalmol..

[31] Bowers A.R., Mandel A.J., Goldstein R.B., Peli E. (2009). Driving with Hemianopia, I: Detection Performance in a Driving Simulator. Investig. Opthalmol. Vis. Sci..

[32] Johnson C.A., Keltner J.L. (1983). Incidence of Visual Field Loss in 20,000 Eyes and Its Relationship to Driving Performance. Arch. Ophthalmol..

[33] Kunimatsu-Sanuki S., Iwase A., Araie M., Aoki Y., Hara T., Nakazawa T., Yamaguchi T., Ono H., Sanuki T., Itoh M. (2015). An assessment of driving fitness in patients with visual impairment to understand the elevated risk of motor vehicle accidents. BMJ Open.

[34] Wood J.M., McGwin G., Elgin J., Vaphiades M.S., Braswell R.A., Decarlo D.K., Kline L.B., Meek G.C., Searcey K., Owsley C. (2009). On-Road Driving Performance by Persons with Hemianopia and Quadrantanopia. Investig. Opthalmol. Vis. Sci..

[35] Bowers A.R. (2016). Driving with homonymous visual field loss: A review of the literature. Clin. Exp. Optom..

[36] Elgin J., McGwin G., Wood J.M., Vaphiades M.S., Braswell R.A., DeCarlo D.K., Kline L.B., Owsley C. (2010). Evaluation of On-Road Driving in People with Hemianopia and Quadrantanopia. Am. J. Occup. Ther..

[37] Parker W.T., McGwin G., Wood J.M., Elgin J., Vaphiades M.S., Kline L.B., Owsley C. (2010). Self-Reported Driving Difficulty by Persons with Hemianopia and Quadrantanopia. Curr. Eye Res..

[38] Kübler T.C., Kasneci E., Rosenstiel W., Heister M., Aehling K., Nagel K., Schiefer U., Papageorgiou E. (2015). Driving with Glaucoma: Task Performance and Gaze Movements. Optom. Vis. Sci..

[39] Coeckelbergh T.R.M., Brouwer W.H., Cornelissen F.W., Kooijman A.C. (2004). Predicting Practical Fitness to Drive in Drivers with Visual Field Defects Caused by Ocular Pathology. Hum. Factors.

[40] Kristalovich L., Ben Mortenson W. (2019). Visual Field Impairment and Driver Fitness: A 1-Year Review of Crashes and Traffic Violations. Am. J. Occup. Ther..

[41] Owsley C., Ball K., McGwin G., Sloane M.E., Roenker D.L., White M.F., Overley E.T. (1998). Visual Processing Impairment and Risk of Motor Vehicle Crash Among Older Adults. JAMA.

[42] International Council of Ophthalmology. Vision requirements for driving safety. Proceedings of the 30th World Ophthalmology Congress.

[43] Rubin G.S., Roche K.B., Prasada-Rao P., Fried L.P. (1994). Visual Impairment and Disability in Older Adults. Optom. Vis. Sci..

[44] Owsley C., Stalvey B.T., Wells J., Sloane M.E., McGwin G. (2001). Visual Risk Factors for Crash Involvement in Older Drivers with Cataract. Arch. Ophthalmol..

[45] Babizhayev M.A., Minasyan H., Richer S.P. (2009). Cataract halos: A driving hazard in aging populations. Implication of the Halometer DG test for assessment of intraocular light scatter. Appl. Ergon..

[46] Kimlin J.A., Black A.A., Wood J.M. (2017). Nighttime Driving in Older Adults: Effects of Glare and Association with Mesopic Visual Function. Investig. Opthalmol. Vis. Sci..

[47] Dakroub M., Boueiri M., Al-Haddad C. (2021). A Review of Driving and Binocularity. J. Pediatr. Ophthalmol. Strabismus.

[48] Baker J.M., Drews-Botsch C., Pfeiffer M.R., Curry A.E. (2019). Driver licensing and motor vehicle crash rates among young adults with amblyopia and unilateral vision impairment. J. Am. Assoc. Pediatr. Ophthalmol. Strabismus.

[49] Valls J.N., Mercado G.R., Tortuero E.G., Moraga A.L., Gene A., Ramos C.S. (2010). Visual acuity of people over 55 years old in habitual corrective conditions: Drivers versus non-drivers. Acta Ophthalmol..

[50] Katsouri I., Athanasiadis L., Bekiaris E., Tsolaki M. (2019). Differences between professional and non-professional drivers with cognitive disorders. Hell. J. Nucl. Med..

[51] Gené-Sampedro A., Alonso F., Sánchez-Ramos C., Useche S.A. (2021). Comparing oculomotor efficiency and visual attention between drivers and non-drivers through the Adult Developmental Eye Movement (ADEM) test: A visual-verbal test. PLoS ONE.

